# Del Nido versus HTK cardioplegia for myocardial protection during adult complex valve surgery: a retrospective study

**DOI:** 10.1186/s12872-021-02411-w

**Published:** 2021-12-18

**Authors:** Lian Duan, Guo-huang Hu, E. Wang, Cheng-liang Zhang, Ling-jin Huang, Yan-ying Duan

**Affiliations:** 1 Department of Cardiovascular Surgery, National Clinical Research Center for Geriatric Disorders, Xiangya Hospital, Central South University, Changsha, China; 2 Department of Surgery, Affiliated Changsha Hospital of Hunan Normal University, Changsha, China; 3 Department of Anesthesiology, Xiangya Hospital, Central South University, Changsha, China; 4 Department of Occupational and Environmental Health, Public Health School, Central South University, Changsha, China

**Keywords:** Single-dose cardioplegia, Return to spontaneous rhythm, ICU stay, Economic, Myocardial protection

## Abstract

**Background:**

Histidine-tryptophan-ketoglutarate (HTK) and del Nido (DN) cardioplegia are intracellular-type and extracellular-type solution respectively, both can provide a long period of myocardial protection with single-dose infusion, but studies comparing the two are rare for adult cardiac surgery. This study aims to evaluate whether DN is suitable for cardioplegia in complex and high-risk valve surgery with long-term cardiac ischemia when compared with HTK.

**Methods:**

The perioperative records of adult patients infused with DN/HTK as a cardioplegic solution who underwent complex valve surgery with an expected myocardial ischaemic duration longer than 90 min between Oct 2018 and Oct 2019 were analysed retrospectively.

**Results:**

Of the 160 patients who received DN/HTK and underwent complex valve surgery, we propensity matched 73 pairs. Both groups achieved satisfactory cardiac arrest effects, and no significant difference was found in their cTnI and CK-MB levels within 12 to 72 h postoperatively. The DN group had a higher rate of return to spontaneous rhythm (0.88 *v* 0.52, *P* < 0.001), a lower frequency of postoperative severe arrythmias (12% *v* 26%, *P* = 0.036), a higher postoperative stroke volume (65 *v* 59 ml, *P* = 0.011) and a higher cardiac output (6.0 *v* 4.9 L/min, *P* = 0.007) as evaluated by echocardiography, fewer transfusions and shorter ICU stays (both *P* < 0.05). The two groups had similar inotrope usage and similar incidences of low cardiac output, morbidities and mortality. Subgroup analysis showed that when the aortic clamping time was greater than 120 min, the advantages of DN were weakened.

**Conclusions:**

DN can be safely applied to complex valve surgery, and it has a similar myocardial protection effect as HTK. Further prospective studies are required to verify these retrospective findings.

*Trial registration* retrospectively registered.

**Supplementary Information:**

The online version contains supplementary material available at 10.1186/s12872-021-02411-w.

## Background

Single-dose cardioplegia during complex, minimally invasive or redo cardiac operations is attractive to surgeons since it avoids interruption of the ongoing procedure and decreases the aortic clamping time [1]. Several studies have found beneficial effects of a longer cardiac ischaemia time with the use of single-dose cardioplegic infusion, such as with histidine-tryptophan-ketoglutarate (HTK) or del Nido (DN) solution [2–4], but both types are often compared with conventional blood cardioplegia (20–30 min interval). DN solution offers 60 to 90 min of arrest before redosing is required [4, 5], and HTK (Custodiol®) may offer even longer protection (more than 2 h) [6].

Both HTK and DN cardioplegia have been applied in adult cardiac surgery for many years, but few studies have directly compared the two therapies. One possible reason is that HTK is an intracellular-type cardioplegia (all crystalloid), while DN is an extracellular-type cardioplegia mixed with blood (crystalloid: blood = 4:1). The formula, induction dose, and reperfusion interval of the two are different. In our literature review, we could only find two published papers that directly compared DN and HTK. One treated paediatric patients undergoing surgical correction for tetralogy of Fallot [7], and the other enrolled adult low-risk patients undergoing minimally invasive cardiac surgery [8]. The two studies demonstrated better outcomes in the DN group for some clinical endpoints. However, the myocardial protective effect of DN for high-risk adult patients or during complex procedures requiring a long arrest is uncertain.We began to use DN cardioplegia in adult complex valve surgery in September 2018 in our hospital. To evaluate the safety, efficacy and myocardial protection effect of DN cardioplegia in adult complex valve surgery, we retrospectively compared this solution with the classic HTK solution.

## Methods

### Patients

The medical records of consecutive adult patients who underwent valve surgery at Xiangya Hospital of Central South University from Oct 2018 to Oct 2019 were analysed retrospectively. The ethics review board of our hospital approved the study (ID:2018081047), and the requirement for informed consent was waived because of the retrospective design. Patients who were older than 16 years and who underwent complex valve surgery were included. “Complex valve surgery” meant elective multiple valve surgery plus at least one of the following procedures: aortic vessel/coronary artery bypass graft/maze/congenital heart defect/cardiac tumour or redo/mini-multiple valve surgery. These procedures have an expected cardiopulmonary bypass duration of more than 150 min and an aortic clamping time of more than 90 min, and require multiple infusions of conventional potassic blood cardioplegia. Conventional potassic blood cardioplegia was our center's strategy for routine single procedures, while HTK or DN for multiple procedures. The following patients were excluded from the analysis: those with incomplete records in electronic medical records, those who were not managed using HTK or DN cardioplegia, those who were managed using two kinds of cardioplegia.

### Cardioplegia

The formula, final ion concentration and method of application are listed in Table 1. According to the different types of cardioplegia used during the operation, patients were divided into two groups: the DN and HTK groups. When DN/HTK solution was infused, at least one check was performed to ensure that there was no incomplete clamping/cardioplegia solution leakage. If residual electrical activity (an electrocardiogram showed no interference but instead showed waves of cardiac activity during cross-clamping) occurred and continued (not intermittently) for more than 3 min, reperfusion was necessary. The reperfusion dosing for residual activity was 200–300 ml or until no electrical activity was present.

**Table 1:** The formula, ions concentration when used, application methods and prices of two kinds of cardioplegia solutions: DN versus HTK

	DN		HTK	
	Extracellular-type		Intracellular-type	
Formula	Plasma-lyte A	500 ml	Water for injection	1000 ml
	10% potassium chloride	10 ml	NaCl	0.8766 g
	5% sodium bicarbonate	11 ml	KCl	0.6710 g
	25% magnesium sulfate	4 ml	MgCl_2_.6H_2_O	0.8132 g
	20% mannitol	8.15 ml	mannitol	5.4651 g
	2% lidocaine	3.25 ml	Histidine	27.9289 g
			Tryptophan	0.4085 g
			Histidine.HCl.H_2_O	3.7733 g
			CaCl_2_.2H_2_O	0.0022 g
			2-Ketoglutarate-H–K	0.1842 g
Ions concentration when used				
Na^+^ (mmol/L)		150		15
Cl^−^ (mmol/L)		132		100
K^+^ (mmol/L)		24		9
Mg^2+^ (mmol/L)		6		4
Ca^2+^ (mmol/L)		0.4		0.02
Lidocaine (mg/L)		140		0
Mannitol (g/L)		2.6		5.46
Histidine (mmol/L)		0		198
Tryptophan (mmol/L)		0		2
Ketoglutarate (mmol/L)		0		1
Application method				
	Induction dose	mixing with 1/4 blood, 20 ml/kg, up to 1000 ml		20–30 ml/kg, up to 2000 ml
	Temperature(°C)	4–15		4
	Pressure(antegrade/retrograde)	140/45		140/45
	Perfusion speed(minutes)	3–5		More than 5
	Reinfusion interval (minutes)	60–90		60–120,even more
	Maintenance dose	200–300 ml		500 ml
	Improvisation by doctors before use		Commercial product, use anytime	
Price	About ¥50/500 ml		About ¥1200/500 ml	

DN, del Nido; HTK, histidine-tryptophan-ketoglutarate

### Perioperative data

All patients were cared for by the same team of anaesthesiologists, surgeons, perfusionists, and intensivists. The details of the anaesthesia, surgical procedure, and cardiopulmonary bypass itself were reported previously [9]. Postoperatively, all patients were transported to the intensive care unit (ICU) while intubated and ventilated. The demographic characteristics, cardiac comorbidity, type of surgery along with intraoperative variables, duration of postoperative mechanical ventilation (MV), length of ICU stay, postoperative hospital stay, and short-term outcomes (postoperative inotropic administration, perioperative blood transfusion, postoperative morbidity and mortality) were recorded. Inotropic administration was defined as the dosage of either dopamine (Dop), epinephrine (E) or norepinephrine (NE), for at least 1 h during the first 24 postoperative hours, and were recorded as grade (Additional file 2: instruction 1). Allogeneic transfusions were performed according to the ‘Granducato algorithm’ [10]: haemoglobin < 7 g/dl, intraoperative evidence of end-organ ischaemia, life-threatening bleeding, and viscoelastic tests for bleeding management. Postoperative morbidity included cardiac (low cardiac output, postoperative severe arrhythmia) and non-cardiac [postoperative multiple organ dysfunction syndrome (MODS), postoperative central nervous system (CNS) injury, postoperative lung injury, postoperative acute kidney injury, postoperative acute liver injury, postoperative stress ulcer] morbidity. Postoperative low cardiac output [11] refers to the need for a high dose of inotropics (Dop > 15 μg kg^−1^ min^−1^/E > 0.1 μg kg^−1^ min^−1^/NE > 0.1 μg kg^−1^ min^−1^) to maintain a systolic blood pressure greater than 90 mmHg or the need for mechanical circulation support [extracorporeal membrane oxygenation (ECMO) or intra-aortic balloon pump (IABP)] after the operation. Severe arrythmia refers to total atrioventricular block, sudden cardiac arrest and ventricular fibrillation. CNS injury [12] refers to new onset cerebral haemorrhage, cerebral infarction, paraplegia, hemiplegia, acute mental disorder or hypoxic-ischaemic encephalopathy (temporary or permanent). Lung injury [13] refers to respiratory failure, new-onset pulmonary embolism/oedema/pneumonia/massive pleural effusion, or hypoxemia exceeding 24 h. Acute kidney injury [14] refers to new-onset renal failure requiring renal replacement or basline creatinine elevated by more than 50%. Acute liver injury [15] refers to new-onset hepatic failure or total bilirubin > 51 μmol/L. Stress ulcer refers to new-onset gastrointestinal haemorrhage or melena. Thirty-day mortality (including patients who died 30 days after the operation but who were not discharged) was recorded. Perioperative laboratory tests [cardiac troponin I (cTnI), creatine kinase myocardial isoenzyme (CK-MB), serum aspartate transaminase (AST)] and some echocardiography data were also recorded.

### Outcomes

The primary outcome was the highest serum cTnI level within postoperative 12 h. Secondary outcomes contained inotropes, incidency of return to spontaneous rhythm after removing aortic clamping forceps, postoperative severe arrythmias, and low cardiac output. Furthermore, stroke volume(SV) and ejection fraction (EF) assessed using echocardiography after surgery, peak value of CK-MB and AST levels on postoperative days 1, 2 and 3 were also included. Tertiary outcomes were the aortic clamping time, echocardiography of atrioventricular size data after surgery, and postoperative clinical outcomes including the MV time, ICU time, transfusions, post-hospital days, in-hospital morbidities, and 30-day mortality.

## Statistical analyses

Categorical variables are summarized as frequencies and percentages, while continuous variables are expressed as the mean ± SD when the data were normally distributed and as the interquartile range (IQR) [P_50_(P_25_,P_75_)] when the data were non-normally distributed. To generate two evenly matched cohorts of patients who received DN or HTK, we propensity score matched patients by using the following pre- and intraoperative variables according to univariate logistic regression and the guidance of statistical experts: sex, age, weight, preoperative cTnI, preoperative EF, preoperative SV, preoperative NYHA grade 3–4, preoperative arrhythmia, EuroSCORE II, preoperative lung diseases, preoperative cardiac infarction, diabetes, combination with aortic surgery, combination with a coronary artery bypass graft (CABG) operation,preoperative serum creatinine, and CPB time. Of the 76 patients who received DN, we matched 73 DN patients with 73 who received HTK patients who underwent complex valve surgery during the same time period. The remaining patients had no suitable propensity scores. The matching tolerance was 0.15, and the propensity scores ranged from 0.12779 to 0.86060. Discrimination and calibration of propensity scores were assessed with the Hosmer–Lemeshow test and C-statistics. After matching, the most of standard mean differences (SMDs) became smaller(see the SMDs in Additional file 2: instruction 2). The chi-square test was used for categorical variables, Mann–Whitney *U* test was used for continuous variables when the data were non-normally distributed, and *t*-test was used when data were normally distributed. Perioperative laboratory tests of myocardial injury markers were compared using analysis of variance of repeated measurement data. Before subgroup analysis, the comparability of pre-operative and some intra-operative variables within each subgroup had been proved by Mann–Whitney *U* test or chi-square test (see Additional file 2: instruction 3). The trial was designed to investigate the potential superiority of DN in terms of decreasing postoperative cardiac injury. According to the previous studies [11, 16], we inputed the mean intervention group and the control group value of cTnI with 7.3 and 8.3 respectively. The standard deviation is 10.8, with difference 4 is clinical significant. The sample calculator outputed that 58 patients per group would be sufficient with a power of 0.8 and an alpha risk of 0.05. A *p* value < 0.05 was considered significant, and *p* < 0.15 was considered to be included in the matching model. Missing data within 10% were filled with multiple imputations. Data analysis was performed using IBM SPSS 23.0 (SPSS Software, IBM Corp., Armonk, NY,USA).

## Results

**Fig. 1:**
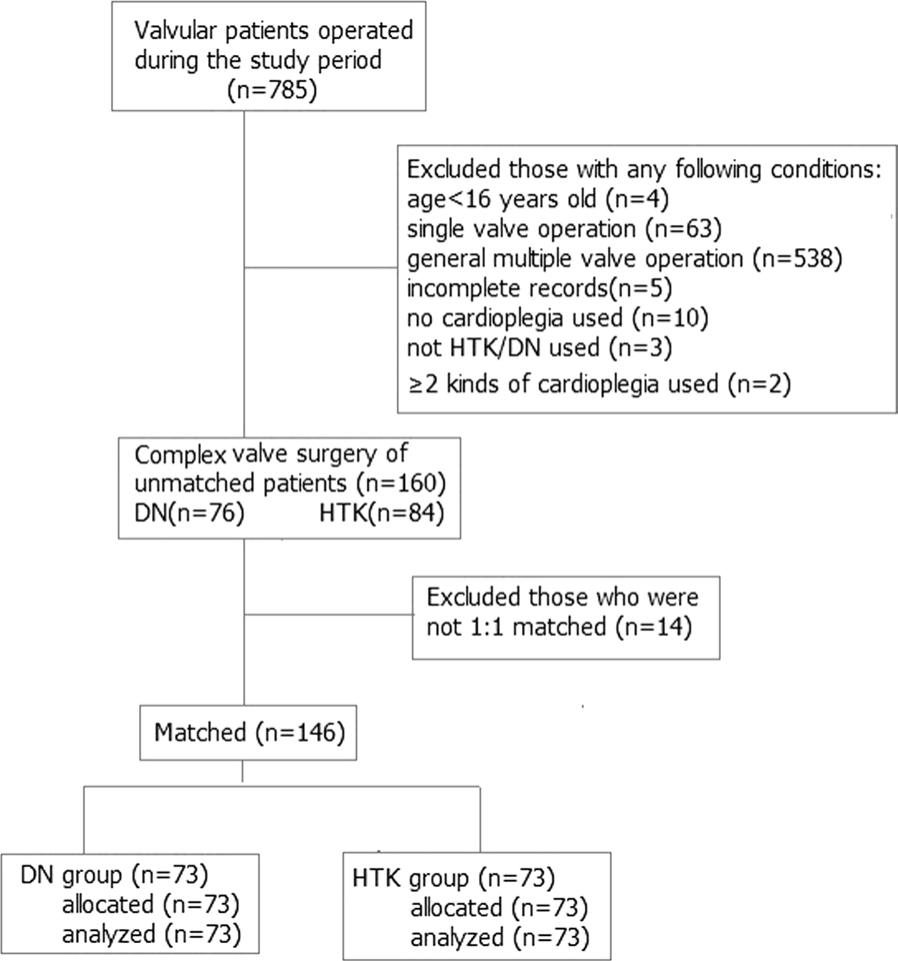
Flow chart diagram

During the study period, 785 patients underwent valvular operation and 146 were matched and analyzed (Fig. 1). The demographic characteristics and preoperative conditions of matched patients were listed in Table 2. All patients were Chinese local residents, the median age was 56 (range 16–77) years, the percentage of male patients exceeded 60% (90/146), and more than half of the patients (77/146) had a small body size (≤ 60 kg, weight range 32–115 kg). As shown in Table 2, most patients had comorbidities impairing recovery in addition to multiple valvular diseases, but there were no significant differences between the two groups.

**Table 2:** Preoperative characteristics of matched patients undergone complex valve surgery

	DN (n = 73)	HTK (n = 73)	*P*-value
Male gender [*n*(%)]	46 (63.0)	44 (60.3)	0.734
Age [*P*_50_(*P*_25_,*P*_75_), year]	56 (47,64)	56 (48,62)	0.677
Weight [*P*_50_(*P*_25_,*P*_75_), kg]	60 (52,65)	61 (54,73)	0.324
BMI	22 (20,25)	23 (21,25)	0.215
Arrhythmia [*n*(%)]	21 (28.8)	19 (26.0)	0.711
Poor cardiac function(NYHA grade3 to 4) [*n*(%)]	26 (35.6)	24 (32.9)	0.727
Previous myocardial infarction [*n*(%)]	1 (1.4)	2 (2.7)	0.560
Previous cardiac surgery [*n*(%)]	9 (12.3)	8 (10.9)	0.796
Coronary artery disease [*n*(%)]	24 (43.8)	25 (34.2)	0.841
Infective endocarditis [*n*(%)]	11 (15.1)	4 (5.5)	0.056
Hypertension [*n*(%)]	23 (31.5)	27 (37.0)	0.485
Diabetes [*n*(%)]	5 (6.8)	6 (8.2)	0.754
Previous stroke [*n*(%)]	7 (9.6)	7 (9.6)	1.000
Pulmonary disease [*n*(%)]	11 (15.1)	11 (15.1)	1.000
Preoperative CK-MB [*P*_50_(*P*_25_,*P*_75_), U/L]	11.0 (8.9,11.1)	13.0 (10,15.1)	0.070
Preoperative cTnI [*P*_50_(*P*_25_,*P*_75_), ng/mL]	0.02 (0.01,0.04)	0.02 (0.01,0.04)	0.727
Preoperative AST [*P*_50_(*P*_25_,*P*_75_), μu/L]	23.0 (18.0,29.3)	22.8 (18.8,31.3)	0.431
Preoperative creatinine [*P*_50_(*P*_25_,*P*_75_), μmol /L]	84.2 (73.1,94.5)	83 (74.8,100.8)	0.418
EuroSCORE II	4.08 (2.94,6.89)	4.30 (2.79,7.51)	0.902

DN, del Nido; HTK, histidine-tryptophan-ketoglutarate; NYHA, the New York Heart Association; CK-MB, creatine kinase myocardial isoenzyme; cTnI, cardiac troponin I; AST, serum aspartate transaminase

### Overall evaluation

The two kinds of cardioplegic solutions both achieved satisfactory cardiac arrest effects. Residual electrical activity occurred in the HTK group(the DN group didn’t) sometimes but non-continued, so reperfusion was not needed. DN was cheaper than HTK but inconvenient (DN was improvisated by doctors and drug-error probability may happen, HTK was a commericial product and can be used anytime). As shown in Table 3, the DN group had a shorter reinfusion interval (50 vs. 83 min, *P* < 0.001) and a higher number of infusions than the HTK group (2 vs. 1, *P* < 0.001). The total cardioplegic volume was lower in the DN group than in the HTK group (1265 vs. 1900 ml, *P* < 0.001). The two groups had similar procedures, cardiopulmonary bypass times and aortic clamping times.

**Table 3:** Perioperative details of paired patients undergone complex valve surgery

	DN (n = 73)	HTK (n = 73)	*P*-value
Valves:aortic + mitral + tricuspid [*n*(%)]	19 (26.0)	16 (21.9)	0.561
Valves:aortic + mitral [*n*(%)]	25 (34.2)	31 (42.5)	0.307
Valves:aortic + tricuspid [*n*(%)]	5 (6.8)	6 (8.2)	0.754
Valves: mitral + tricuspid [*n*(%)]	22 (30.1)	18 (24.7)	0.458
Valves:pulmonary + tricuspid [*n*(%)]	2 (2.7)	2 (2.7)	1.000
Combined with aorta operation [*n*(%)]	16 (21.9)	20 (27.4)	0.442
Combined with CABG operation [*n*(%)]^	24 (32.9)	25 (34.2)	0.861
Combined with others# operation [*n*(%)]	30 (41.1)	26 (35.6)	0.496
Aortic valve mechanic [*n*(%)]	23 (31.5)	20 (27.4)	0.586
Cardiopulmonary bypass time [*P*_50_(*P*_25_,*P*_75_), min]	149 (116,181)	158 (126,187)	0.232
Cardioplegia: numbers of infusion [*P*_50_(*P*_25_,*P*_75_)]	2 (1,3)	1 (1,2)	0.000
Cardioplegia: infusion interval time [*P*_50_(*P*_25_,*P*_75_), min]	50 (43,63)	83 (60,95)	0.000
Cardioplegia: antegrade infusion [*n*(%)]	67 (91.8)	70 (95.9)	0.302
Cardioplegia: volume [*P*_50_(*P*_25_,*P*_75_), mL]	1265 (1000,1500)	1900 (1500,2000)	0.000
Aortic clamping time [*P*_50_(*P*_25_,*P*_75_), min]	107 (74,134)	100 (83,133)	0.795
Return to spontaneous rhythm [*n*(%)]	64 (87.7)	38 (52.1)	0.000
MV time [*P*_50_(*P*_25_,*P*_75_), hour]	15 (11,25)	19 (14,27)	0.131
ICU stay [*P*_50_(*P*_25_,*P*_75_), hour]	29 (20,68)	43 (21,75)	0.024
Post-hospital stay [*P*_50_(*P*_25_,*P*_75_), day]	8 (7,11)	9 (7,11)	0.933
Dop [*P*_50_(*P*_25_,*P*_75_)]	2 (1,3)	3 (1,4)	0.341
Ne [*P*_50_(*P*_25_,*P*_75_)]	3 (1,3)	3 (2,4)	0.153
E [*P*_50_(*P*_25_,*P*_75_)]	1 (0,2)	1 (0,3)	0.161
Transfusion: platelet [*P*_50_(*P*_25_,*P*_75_), unit]	0 (0,0)	0 (0,1)	0.000
Transfusion: erythrocyte [*P*_50_(*P*_25_,*P*_75_), unit]	0.75 (0,4)	0 (0,4)	0.682
Transfusion: plasma [*P*_50_(*P*_25_,*P*_75_), unit]	4 (0,7)	5 (3,9)	0.034
Transfusion: cryoprecipitate [*P*_50_(*P*_25_,*P*_75_), unit]	0 (0,0)	0 (0,16)	0.031
Mortality [*n*(%)]	3 (4.1)	6 (8.2)	0.302
Postoperative low cardiac output [*n*(%)]	6 (8.2)	12 (16.4)	0.131
Postoperative arrhythmia [*n*(%)]	9 (12.3)	19 (26.0)	0.036
Postoperative AKI [*n*(%)]	18 (24.7)	20 (27.4)	0.706
Postoperative ALI [*n*(%)]	3 (4.1)	2 (2.7)	0.649
Postoperative MODS [*n*(%)]	4 (7)	5 (7)	0.731
Postoperative lung injury [*n*(%)]	19 (26.0)	19 (26.0)	1.000
Postoperative CNS inury [*n*(%)]	3 (4.1)	2 (2.7)	0.649
Postoperative stress ulcer [*n*(%)]	1 (1.4)	3 (4.1)	0.311

Others #: maze/congenital heart defect/cardiac tumor, or redo/mini procedures.(DN/HTK was 11/9 in maze, 5/2 in congenital heart defect, 1/5 in cardiac tumor, 6/4 in redo, 7/7 in mini/laparoscopic procedures, respectively)

DN, del Nido; HTK, histidine-tryptophan-ketoglutarate; CABG, coronary artery bypass graft; MV, mechanical ventilation; ICU, intensive care unit; Dop, dopamine; NE, norepinephrine; E, epinephrine; AKI, acute kidney injury; ALI, acute liver injury; MODS, multiple organ dysfuntion syndrome; CNS, central nervous system

### Outcomes

**Fig. 2:**
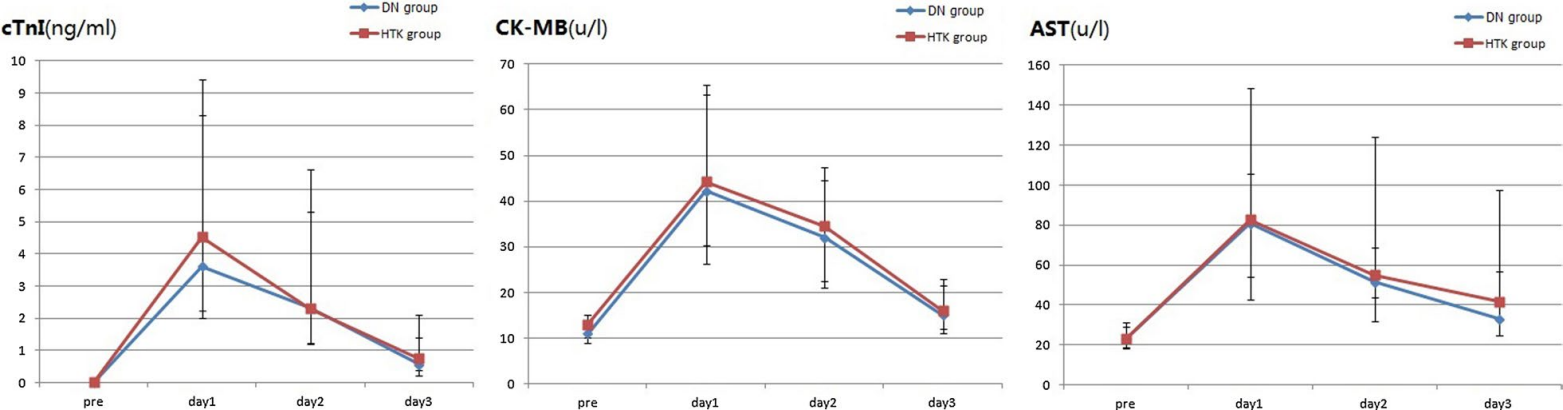
Perioperative comparison serum levels of myocardial injury markers between the DN group and the HTK group. DN, del Nido; HTK, histidine-tryptophan-ketoglutarate; cTnI, cardiac troponin I; CK-MB, creatine kinase myocardial isoenzyme; AST, serum aspartate transaminase. (See the Additional file 2: instruction 4, no statistical differences were found)

As shown in Fig. 2, Tables 3 and 4, no significant difference was found between the two groups in terms of the peak cTnI,CK-MB or AST levels on postoperative days 1, 2 and 3. There was also no differences for postoperative inotropes, EF, the incidence of low cardiac output, the aortic clamping time, the atrioventricular size on echocardiography, MV time, postoperative hospital days, in-hospital morbidities, or 30-day mortality. However, in the DN group, there were a higher incidence of return to spontaneous rhythm and a lower incidences of postoperative severe arrythmias, more SV and CO after surgery, a shorter ICU time and fewer transfusions.

**Table 4:** Perioperative results of echocardiography in paired patients undergone complex valve surgery

	Pre-operative			Post-operative			
	DN (n = 73)	HTK (n = 73)	*P*-value	DN (n = 73)		HTK (n = 73)	*P*-value
EF [*P*_50_(*P*_25_,*P*_75_),%]	58 (53,64)	59 (50,67)	0.481	57 (49,62)		54 (45,62)	0.261
SV [*P*_50_(*P*_25_,*P*_75_),ml]	85 (71,111)	81 (67,103)	0.128	65 (52,85)		59 (45,73)	0.011
CO [*P*_50_(*P*_25_,*P*_75_), L/min]	6.7 (5.5,9.2)	6.2 (4.9,8.7)	0.116	6.0 (4.7,7.1)		4.9 (3.7,6.5)	0.007
HR [*P*_50_(*P*_25_,*P*_75_),/min]	78 (68,90)	78 (69,91)	0.801	91 (79,103)		86 (77,98)	0.619
FS [*P*_50_(*P*_25_,*P*_75_), %]	31 (27,35)	31 (26,37)	0.826	30 (24,33)		28 (22,33)	0.279
LV size [*P*_50_(*P*_25_,*P*_75_), mm]	56 (51,62)	55 (46,65)	0.526	50 (45,55)		49 (45,55)	0.575
RV size [*P*_50_(*P*_25_,*P*_75_),mm]	18 (16,20)	18 (16,21)	0.742	18 (16,19)		17 (15,20)	0.955
4CV* RV size [*P*_50_(*P*_25_,*P*_75_), mm]	34 (31,36)	34 (31,38)	0.323	34.0 (31,37)		33 (30,36)	0.235
LA size [*P*_50_(*P*_25_,*P*_75_),mm]	42 (37,50)	42 (37,50)	0.704	37 (32,42)		38 (34,47)	0.099
RA size 1# [*P*_50_(*P*_25_,*P*_75_),mm]	45 (40,51)	45 (41,51)	0.406	43 (39,49)		44 (41,50)	0.167
RA size 2## [*P*_50_(*P*_25_,*P*_75_),mm]	40 (37,45)	40 (36,47)	0.553	40 (36,45.0)		41 (37,45)	0.726

DN, del Nido; HTK, histidine-tryptophan-ketoglutarate;CO, cardiac output; EF, ejection fraction; SV, stroke volume; LV, left ventricle; RV, right ventricle; LA, left atrium; RA, right atrium; FS, fraction shortening;HR, heart rate

^*^4CV, four chamber ventricle cutting surface;

^#^RA1, upper and lower diameter of right atrium in four chamber ventricle cutting surface;

^##^RA2, left and right diameter of right atrium in four chamber ventricle cutting surface

We performed subgroup analyses according to whether the aortic clamping time was greater than 120 min (Table 5). (The baseline and some intra-operative characteristics of subgroup patients were showed in Additional file 2: instruction 3) There were still no significant differences in cTnI and CK-MB (Additional file 1: Fig. S1). In the subgroup < 120 min, less norepinephrine was used in the DN group. In the subgroup ≥ 120 min, postoperative severe arrhythmia, ICU stay, postoperative SV and CO, and transfusion of plasma and cryoprecipitate were no longer significantly different.

**Table 5:** Subgroup analysis of two kinds of cardioplegia solution according to whether the aortic clamping time more than 120 min

Outcomes	Aortic Clamping time					
	≥ 120 min			< 120 min		
	DN(n = 29)	HTK(n = 26)	*P*-value	DN(n = 44)	HTK(n = 47)	*P*-value
Aortic clamping time [*P*_50_(*P*_25_,*P*_75_), min]	137(125,160)	146.5(131,164)	0.194	78(63,102)	87(68,99)	0.432
Return to spontaneous rhythm [*n*(%)]	27(93.1)	11(42.3)	0.000	37(84.1)	27(57.4)	0.005
Dop [*P*_50_(*P*_25_,*P*_75_)]	3(0,3.5)	3(1,3)	0.696	2(1,3)	3(0,4)	0.373
Ne [*P*_50_(*P*_25_,*P*_75_)]	3(1.5,4)	2.5(2,3)	0.620	2(1,3)	3(2,4)	0.025
E [*P*_50_(*P*_25_,*P*_75_)]	2(0,2.5)	2(1,3)	0.437	1(0,2)	1(0,3)	0.233
Postoperative low cardiac output [*n*(%)]	4(13.8)	6(23.1)	0.373	2(4.5)	6(12.8)	0.166
Postoperative arrhythmia [*n*(%)]	5(17.2)	9(34.6)	0.140	4(9.1)	10(21.3)	0.107
MV time [*P*_50_(*P*_25_,*P*_75_), hour]	16(11.5,27.5)	19(13.5,27)	0.813	15(10,21.5)	19(13,26)	0.065
ICU stay [*P*_50_(*P*_25_,*P*_75_), hour]	39(19.5,70)	43.5(19,75.5)	0.906	21(20,43)	42(22,68)	0.004
Post-hospital stay [*P*_50_(*P*_25_,*P*_75_), day]	10(6.5,11.5)	10(7,12)	0.799	8(7,11)	9(7,11)	0.773
Transfusion: platelet [*P*_50_(*P*_25_,*P*_75_), unit]	0(0,0)	0(0,1)	0.042	0(0,0)	0(0,1)	0.000
Transfusion: erythrocyte [*P*_50_(*P*_25_,*P*_75_), unit]	1.5(0,5.75)	2(0,4)	0.799	0(0,2)	0(0,4)	0.549
Transfusion: plasma [*P*_50_(*P*_25_,*P*_75_), unit]	5.5(3,11.5)	7(4,11.5)	0.261	3.5(0,5)	4.5(3,7)	0.037
Transfusion: cryoprecipitate [*P*_50_(*P*_25_,*P*_75_), unit]	0(0,8)	0(0,17.5)	0.354	0(0,0)	0(0,0)	0.028
Mortality [*n*(%)]	2(6.9)	3(11.5)	0.550	1(2.2)	3(6.4)	0.339
Post-operative EF [*P*_50_(*P*_25_,*P*_75_),%]	54(44.5,60.5)	51.5(42,62)	0.933	58.5(53,63)	54(45,61)	0.140
Post-operative SV[*P*_50_(*P*_25_,*P*_75_),ml]	67(50,91.5)	65(51.5,82)	0.418	64.5(54,84)	56(40,66)	0.007
Post-operative CO [*P*_50_(*P*_25_,*P*_75_), L/min]	6.8(4.4,7.7)	6(3.9,7.6)	0.522	5.9(4.8,7.0)	4.8(3.6,6.4)	0.002
Post-operative HR [*P*_50_(*P*_25_,*P*_75_),/min]	91(82,97)	87(78,100)	0.899	90.5(75,104)	86(77,98)	0.551
Post-operative FS [*P*_50_(*P*_25_,*P*_75_), %]	28(22.5,32)	27(21.5,32)	0.692	30(27,34)	28(21,33)	0.294

*DN* del Nido, *HTK* histidine-tryptophan-ketoglutarate, *Dop* dopamine, *NE* norepinephrine, *E* epinephrine, *MV* mechanical ventilation, *ICU* intensive care unit, *EF* ejection fraction, *SV* stroke volume, *CO* cardiac output, *HR* heart rate, *FS* fraction shortening

## Discussion

The main contribution of this study was that DN, when compared with HTK, could be safely and effectively applied during adult complex valve surgery with a long period of myocardial protection. DN had some advantages, such as a higher rate of return to spontaneous rhythm after declamping, fewer transfusions, shorter ICU stays, and increased postoperative SV and CO as assessed by echocardiography. However, when the aortic clamping time was longer than 120 min, the advantages of DN were weakened. Two previous studies directly compared DN and HTK for paediatric use and low-risk adults [7, 8], and we expanded the application of DN to longer aortic clamping times in complex and high-risk procedures.

As a type of extracellular cardioplegia, DN facilitates myocardial contractile arrest by providing a high concentration of potassium ions in the extracellular space mixed with blood (crystalloid: blood = 4:1), while HTK is an intracellular-type cardioplegia, and the arrest mechanism involves the balance of intra- and extracellular sodium ions, requiring a longer infusion period (5–7 min, *v* DN 3–5 min) to reach this balance. Thus, the dosing of DN was less (up to 1000 ml if the weight exceeded 50 kg)[17, 18] than HTK (up to 2000 ml), because the decreased volume and time of perfusion of HTK would result in the development of complications associated with inadequate cardioplegic myocardial protection [19]. Furthermore, the difference in infusion interval time led to a larger number of infusions in the DN group, but it did not affect the aortic clamping time.

The rate of return to spontaneous rhythm in the DN group was higher than that in the HTK group, which was consistent with previous DN cardioplegia versus blood cardioplegia studies [4, 11, 20] (such as modified St. Thomas or Buckberg, blood: crystal = 4:1). The higher rate of return to spontaneous rhythm of DN, which possibly signifies less myocardial ischaemic-reperfusion injury, and the mechanisms are thought to be due to two aspects: the inhibition of calcium influx [21] and the association between the ischaemic myocardium and progressive rewarming during reperfusion [11]. HTK is a pure crystalloid solution with no blood composition; which contains a very low dose of calcium, also contains the Ca^2+^ blocker magnesium sulphate as in DN. However, HTK does not contain lidocaine (another Ca^2+^ blocker);perhaps, the co-effect of blocking calcium accumulation in DN (lidocaine and magnesium) is stronger than that in HTK (only magnesium). Similarly, in recent published articles, the use of HTK was associated with increased reperfusion fibrillation [8, 22].

The reasons for the less frequent postoperative severe arrythmias and the shorter ICU stay in the DN group would be multifactorial: electrolyte disturbance, body temperature, postoperative myocardial ischaemia or edema, neuroendocrine hormone, drugs, etc. Although hyponatremia is more common in HTK, postoperative severe arrythmia is rarely reported. When compared with conventional blood cardioplegia, one study showed that more postoperative atrial fibrillation occurred in DN [23] in the high-risk subgroup analysis. In a recent meta-analysis comparing DN and conventional blood cardioplegia undergoing valve or CABG surgery, the use of DN was associated with a shorter ICU stay [24]. We did not collect eletrolyte, body temperature or neuroendocrine data. After adjusting for some perioperative factors (Additional file 2: instruction 5), the cardioplegia type was no longer a significant influencing factor, but postoperative low cardiac output was. In addition, when we performed subgroup analysis (Table 5), the difference in postoperative arrhythmia was no longer significant.

Echocardiography is the most commonly used noninvasive method for the assessment of cardiac function. Postoperatively, the SV and CO were better in the DN group (see Table 4). Subgroup analysis showed that when the aortic clamping time was longer than 120 min, the differences were no longer significant(Table 5). It was deemed inadequate to predict and monitor myocardial damage by the echocardiographic evaluation of SV/CO because an increase in SV/CO represents a real increase in cardiac function or an increase in extracellular fluid volume. Blood volume increased by 40%, and SV/CO increases by 30% [25]. On the other hand, the presence of artefacts from the mechanical aortic valve made it challenging to measure the left ventricular outflow tract (LVOT) velocity and diameter correctly, which could lead to deviation of the SV/CO measurement [26]. [In our cases, the ratios of the aortic mechanical valve were 31.5% (DN:23/73) vs.27.4% (HTK:20/73), with no significant difference].

Interestingly, the units of erythrocyte administered were similar, but fewer units of platelets,plasma and cryoprecipitate were administered in the DN group. One reason that the cardioplegia type may not influence erythrocyte transfusion may be intraoperative ultrafiltration (all of the patients). We examined some factors affecting platelet transfusions that we did not pay attention to in this study, such as age over 70 years, clopidogrel administration within 7 days before the operation [27], and application of autologous platelet pheresis [28], and no significant difference was found between the two groups (Additional file 2: instruction 6). When the aortic clamping time was longer than 120 min, the transfusion-saving effect of DN was weakened (Table 5), suggesting that additional reinfusions of DN resulted in a higher total cardioplegic volume, dilution and edema as a result of more transfusions [29, 30].

The limitations of our study were its retrospective nature, the limited sample size, the single-centre and single race study, and procedures with CABG or maze could influence the outcome of cTnI. In addition, we did not consider the different temperature of the heart between the two groups or measure the myocardial temperature. On the other hand, we did not calculate the myocardial mass or fluid overload would cause volume bias of cardioplegia and fluid to some extent. Furthermore, more than half of the patients had a small body size, and ultrafiltration used to reduce blood dilution by cardioplegia fluid might make the conclusion of this article not applicable to most patients in Europe and America. The limited sample size was too small to evaluate for adverse morbidity and mortality. We deemed a prospective randomized controlled trial would more better, but the supply of HTK solution from abroad during the COVID-19 epidemic period is very difficult for us. However, we tried our best to provide a comprehensive evaluation of postoperative cardiac function, such as myocardial injury markers, inotropic usage, the rate of return to spontaneous rhythm, echocardiography, incidence of postoperative low cardiac output and severe arrythmias, resource utilization, and heart-related morbidity and mortality. We used Table 6 to summarize advantages and disadvantages of each other. DN was inconvenient (improvisation by doctors when needed) but has an attractive lower cost (shortened ICU stay, fewer transfusions and no expensive drugs in the formula). In developing countries such as China, the labour cost is low, and HTK depends on whole cold-chain transportation or import from abroad, which increase its price. Therefore, we believe that DN is very suitable for underdeveloped regions as long as it is not inferior to HTK.

**Table 6:** Application analysis of two kinds of cardioplegia solution

	DN	HTK
Myocardial injury markers	=	=
Rate of return to spontaneous rhythm	✔	
Aortic clamping time	=	=
Clinical safety	=	=
Economic	✔	
Convenience		✔
Protection time of single dose	60–90 min	60–120 min or more
Echocardiography	=	=

*DN* del Nido, *HTK* histidine-tryptophan-ketoglutarate

## Conclusion

DN can be safely applied to complex valve surgery, and it has a similar myocardial protection effect as HTK. Additional prospective randomised controlled trials comparing the myocardial protective effects of DN and HTK cardioplegia for complex valve surgery are required to verify these retrospective findings.

## Supplementary Information

**Additional file 1: Figure S1**. Subgroup analysis of myocardial injury markers between the DN group and the HTK group according to whether the aortic clamping time was greater than 120 min.

**Additional file 2:** **Supplementary instructions 1–6:** 1. Inotropic grade; 2. SMDs before and after matching; 3. Pre-operative and some intra-operative characteristics of Subgroup patients; 4. Analysis of variance of repeated measurement data; 5. Adjusting some factors affecting postoperative severe arrhythmia; 6. Factors affecting platelet transfusion and adjusting these factors.

## Data Availability

The datasets used and/or analysed during the current study are available from the corresponding author on reasonable request.

## Abbreviations

DN: Del Nido
HTK: Histidine-tryptophan-ketoglutarate
CABG: Coronary artery bypass graft
MV: Mechanical ventilation
ICU: Intensive care unit
Dop: Dopamine
NE: Norepinephrine
E: Epinephrine
ECMO: Extracorporeal membrane oxygenation
IABP: Intra-aortic balloon pump
AKI: Acute kidney injury
ALI: Acute liver injury
MODS: Multiple organ dysfuntion syndrome
CNS: Central nervous system
NYHA: The New York Heart Association
CO: Cardiac output
EF: Ejection fraction
SV: Stroke volume
LV: Left ventricle
RV: Right ventricle
LA: Left atrium
RA: Right atrium
FS: Fraction shortening
HR: Heart rate
