# Supplementary material for: Del Nido versus HTK cardioplegia for myocardial protection during adult complex valve surgery: a retrospective study
Source: BMC Cardiovasc Disord. 2021 Dec 18;21:604. doi: 10.1186/s12872-021-02411-w (PMC8683821; doi:10.1186/s12872-021-02411-w)
Supplement: Supplementary file 1 — Additional file 1: Figure S1. Subgroup analysis of myocardial injury markers between the DN group and the HTK group according to whether the aortic clamping time was greater than 120 min. [file 12872_2021_2411_MOESM1_ESM.docx]

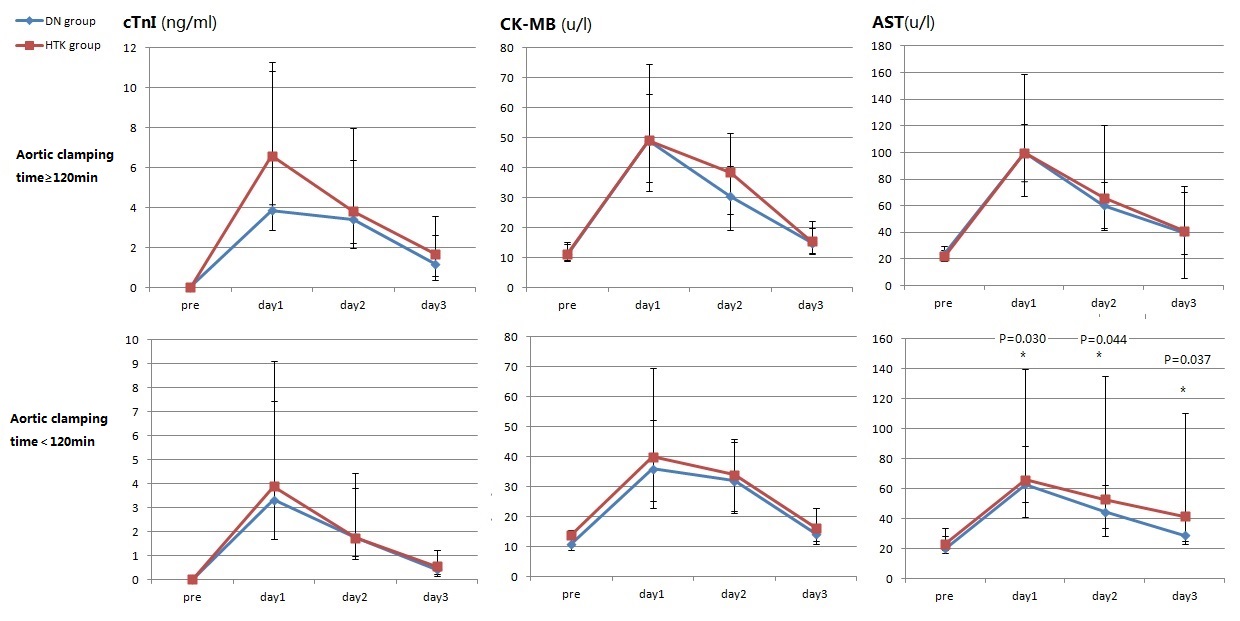


**Figure S1** subgroup analysis of myocardial injury markers between the DN group and the HTK group according to whether the aortic clamping time was greater than 120 min

* *P*<0.05
