# Supplementary material for: Del Nido versus HTK cardioplegia for myocardial protection during adult complex valve surgery: a retrospective study
Source: BMC Cardiovasc Disord. 2021 Dec 18;21:604. doi: 10.1186/s12872-021-02411-w (PMC8683821; doi:10.1186/s12872-021-02411-w)
Supplement: Supplementary file 2 — Additional file 2: Supplementary instructions 1–6: 1. Inotropic grade; 2. SMDs before and after matching; 3. Pre-operative and some intra-operative characteristics of Subgroup patients; 4. Analysis of variance of repeated measurement data; 5. Adjusting some factors affecting postoperative severe arrhythmia; 6. Factors affecting platelet transfusion and adjusting these factors. [file 12872_2021_2411_MOESM2_ESM.docx]

Instruction 1

Inotropic grade

|  | 0 | 1 | 2 | 3 | 4 |
| --- | --- | --- | --- | --- | --- |
| Dopamine(μg/kg/min) | None | <5 | 5-10 | 10-15 | >15 |
| Norepinephrine(μg/kg/min) | None | <0.1 | 0.1-0.2 | 0.2-0.5 | >0.5 |
| Epinephrine(μg/kg/min) | None | <0.1 | 0.1-0.5 | >0.5 |  |

Instruction 2

SMDs before and after matching


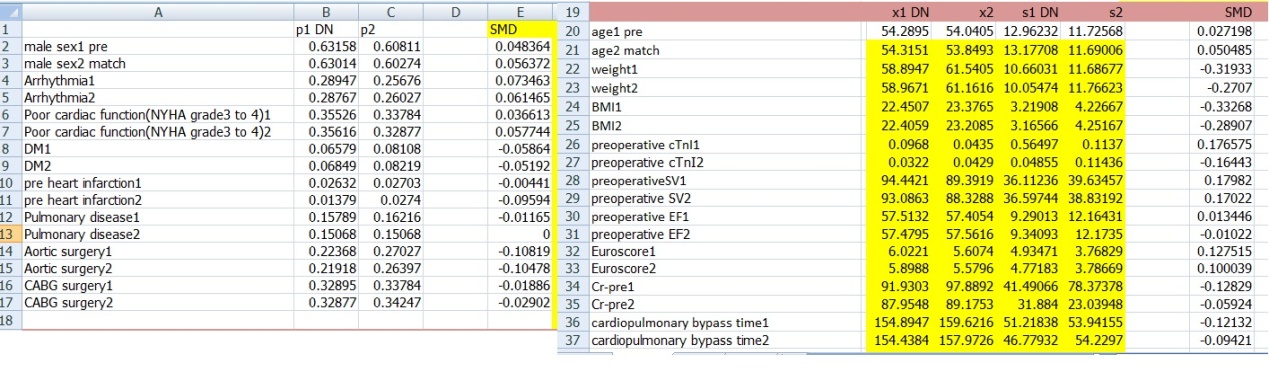


Instruction 3

Pre-operative and some intra-operative characteristics of Subgroup patients

| Aortic clamping time  Outcomes | ≥120 minutes | | | <120 minutes | | |
| --- | --- | --- | --- | --- | --- | --- |
|  | DN(n=29) | HTK(n=26) | *P*-value | DN(n=44) | HTK(n=47) | *P*-value |
| Male gender [*n*(%)] | 19(65.5) | 16(61.5) | 0.759 | 27(61.4) | 28(59.6) | 0.862 |
| Age [*P*_50_(*P*_25_,*P*_75_), year] | 56(48,67) | 55(47,64) | 0.607 | 52(41,61) | 56(48,63) | 0.097 |
| Weight [*P*_50_(*P*_25_,*P*_75_), kg] | 58(52,64) | 61(54,65) | 0.643 | 61(53,67) | 60(53,70) | 0.311 |
| BMI | 22(19,25) | 22(20,24) | 0.913 | 23(20,24) | 24(21,26) | 0.101 |
| Preoperative cTnI [*P*_50_(*P*_25_,*P*_75_), ng/mL] | 0.03(0.01,0.05) | 0.02(0.01,0.03) | 0.221 | 0.02(0.01,0.03) | 0.02(0.01,0.04) | 0.646 |
| Pre-operative EF[*P*_50_(*P*_25_,*P*_75_),%] | 56(50,64) | 57(49,64) | 0.987 | 59(55,65) | 62(55,68) | 0.388 |
| Pre-operative SV[*P*_50_(*P*_25_,*P*_75_),ml] | 93(70,115) | 88(75,128) | 0.953 | 84(71,109) | 76(62,98) | 0.065 |
| Poor cardiac function(NYHA grade 3 to 4) [*n*(%)] | 13(44.8) | 7(26.9) | 0.168 | 13(29.5) | 17(36.2) | 0.502 |
| Pre-operative arrhythmia [*n*(%)] | 12(41.4) | 9(34.6) | 0.606 | 9(37.5) | 10(21.3) | 0.923 |
| EuroSCORE II | 4.73(3.22,7.95) | 4.19(2.92.8.68) | 0.762 | 3.78(2.83,6.29) | 4.25(2.68,7.38) | 0.871 |
| Pulmonary disease[*n*(%)] | 3(10.3) | 4(15.4) | 0.576 | 8(33.3) | 7(14.9) | 0.673 |
| Previous myocardial infarction[*n*(%)] | 1(3.4) | 0(0) | 0.339 | 0(0) | 2(4.3) | 0.166 |
| Diabetes[*n*(%)] | 3(10.3) | 0(0) | 0.092 | 2(4.5) | 6(12.8) | 0.166 |
| Combined with aorta operation [*n*(%)] | 5(17.2) | 8(30.8) | 0.238 | 11(25.0) | 12(25.5) | 0.953 |
| Combined with CABG operation [*n*(%)] | 17(58.6) | 10(38.5) | 0.135 | 7(15.9) | 15(31.9) | 0.075 |
| Preoperative creatinine [*P*_50_(*P*_25_,*P*_75_), μmol /L] | 86(77,102) | 92(77,115) | 0.479 | 81(68,90) | 80(74,100) | 0.494 |
| Cardiopulmonary bypass time [*P*_50_(*P*_25_,*P*_75_), min] | 181(163,217) | 193(174,235) | 0.206 | 127(106,142) | 134(115,159) | 0.124 |

DN, del Nido; HTK, histidine-tryptophan-ketoglutarate;BMI, body mass index; cTnI, cardiac troponin I; EF, ejection fraction; SV, stroke volume; NYHA, the New York Heart Association; CABG, coronary artery bypass graft.

Instruction 4

Analysis of variance of repeated measurement data

| variables | Time *P* | Time×T0 *P* | Time×Group *P* | Group *P* |
| --- | --- | --- | --- | --- |
| cTnI | 0.000 | 0.819 | 0.615 | 0.189 |
| CK-MB | 0.052 | 0.780 | 0.217 | 0.099 |
| AST | 0.491 | 0.854 | 0.293 | 0.447 |

Instruction 5

After adjusting some factors affecting postoperative severe arrhythmia, the cardioplegia type was no longer the significant influence factor in Logistic regression model.

| Variables | OR(95%CI) | *P* value |
| --- | --- | --- |
| Cardioplegia type(DN/HTK) | 2.136(0.711-6.420) | 0.176 |
| Coronary artery disease | 0.805(0.139-4.661) | 0.809 |
| EuroSCORE II | 1.055(0.942-1.182) | 0.351 |
| Preoperative CK-MB | 1.105(1.002-1.217) | 0.045 |
| Aortic clamping time | 1.008(0.996-1.019) | 0.196 |
| Ne | 0.966(0.585-1.595) | 0.891 |
| E | 1.212(0.746-1.969) | 0.437 |
| Postoperative low cardiac output | 0.06(0.013-0.272) | 0.000 |

DN, del Nido; HTK, histidine-tryptophan-ketoglutarate; CK-MB, creatine kinase myocardial isoenzyme; NE, norepinephrine; E, epinephrine.

Instruction 6

Comparion of of paired patients undergone complex valve surgery on factors affecting platelet transfusion

|  | DN(n=73) | HTK(n=73) | *P* value |
| --- | --- | --- | --- |
| age over 70 years[*n*(%)] | 6(8.2) | 4(5.5) | 0.512 |
| clopidogrel administration[*n*(%)] | 6(8.2) | 7(9.6) | 0.771 |
| autologous platelet pheresis[*n*(%)] | 5(6.8) | 12(16.4) | 0.071 |

After adjusting some factors affecting platelet transfusion, the cardioplegia type was still the significant influence factor in Linear regression models.

|  | Variables | B(95%CI) | *P* value |
| --- | --- | --- | --- |
| Model 1 | Cardioplegia type(DN /HTK) | -0.315(-0.470,-0.160) | <0.001 |
| Model2 | Cardioplegia type(DN /HTK) | -0.248(-0.470,-0.160) | <0.001 |
|  | Age over 70 years | 0.059(-0.195,0.313) | 0.647 |
|  | Clopidogrel administration | 0.656(0. 425-0.887) | <0.001 |
|  | Autologous platelet pheresis | 0.247(-0.001-0.494) | 0.051 |
|  | Previous cardiac surgery | 0.200(0.001-0.399) | 0.049 |
|  | Combined with aorta operation | 0.271(0.072-0.469) | 0.008 |
|  | Cardiopulmonary bypass time | 0.002(0.000-0.003) | 0.020 |
|  | Preoperative LA size | 0.007(0.001-0.012) | 0.028 |

DN, del Nido; HTK, histidine-tryptophan-ketoglutarate; LA, left atrium.
